# Ventricular Septal Perforation after Biventricular Takotsubo Cardiomyopathy Successfully Repaired with an Amplatzer Device: First Report in the Literature

**DOI:** 10.1155/2016/3251032

**Published:** 2016-04-13

**Authors:** Alfredo E. Rodríguez, Carlos Fernandez-Pereira, Juan Mieres, Diego Ascarrunz, Eduardo Gabe, Alfredo Matías Rodríguez-Granillo, Romina Frattini, Pablo Stuzbach

**Affiliations:** ^1^Cardiac Unit, Sanatorio Otamendi, Callao 1441 4B, 1024AAA Buenos Aires, Argentina; ^2^Instituto Cardiovascular San Isidro, Las Lomas, Buenos Aires, Argentina

## Abstract

A 79-year-old female was admitted with sudden onset dyspnea, mild oppressive chest pain, and severe anxiety disorder. Patient had history of hypertension, dyslipidemia, smoking, and chronic obstructive pulmonary disease. On admission blood pressure was 160/90 and heart rate was 130 bpm. Transthoracic echocardiography (TE) and contrast tomography showed a thin septum with an abnormal left and right ventricular contraction with an “apical ballooning” pattern and mild increase of cardiac enzymes. At the 4th day of admission, the patient presented symptoms and signs of congestive heart failure and developed cardiogenic shock. EKG showed an inversion of T waves in all precordial leads. In a new TE, a ventricular septal perforation (VSP) in the apical portion of the septum was seen. Coronary angiogram showed angiographically “normal” coronary arteries. With a diagnosis of VSP in takotsubo cardiomyopathy, a percutaneous procedure to repair the VSP was performed 11 days after admission. The VSP was closed with an Amplatzer device. TE performed 24 hours after showed significant improvement of ventricular function and good apposition of the Amplatzer device. Three days later she was discharged from the hospital. To our knowledge, this is the first reported case of a VSP in a TCM repaired percutaneously with an occluder device.

## 1. Introduction

Takotsubo cardiomyopathy (TCM) was characterized by transient left ventricular dysfunction usually involving anteroapical and inferoapical regions of the myocardium in the absence of significant coronary artery disease in the majority of patients [1, 2].

It was first considered to have a good prognosis; however, some TCM patients also developed severe cardiac complications including cardiac rupture (CR) in 0.2% [2, 3].

Hereby, we are reporting a TCM patient with a ventricular septal perforation (VSP) successfully repaired with an Amplatzer occluder device and to our knowledge this is the first reported case in the literature using this percutaneous technique.

## 2. Case Report

A 79-year-old female was admitted to our hospital with sudden onset dyspnea, mild oppressive chest pain, severe anxiety disorder, and the use of the accessory respiratory muscles. The patient had a history of hypertension, dyslipidemia, smoking, chronic obstructive pulmonary disease, and chronic atrial fibrillation under anticoagulation therapy. Physical examination showed coarse crackles in the lower two-thirds of the lungs, wheezing, tachycardia with irregular rate, and no cardiac gallop or murmurs. Blood pressure (BP) was 160/90 hpm, irregular pulse was 130, and respiratory rate was 30 per minute.

**Figure 1:**
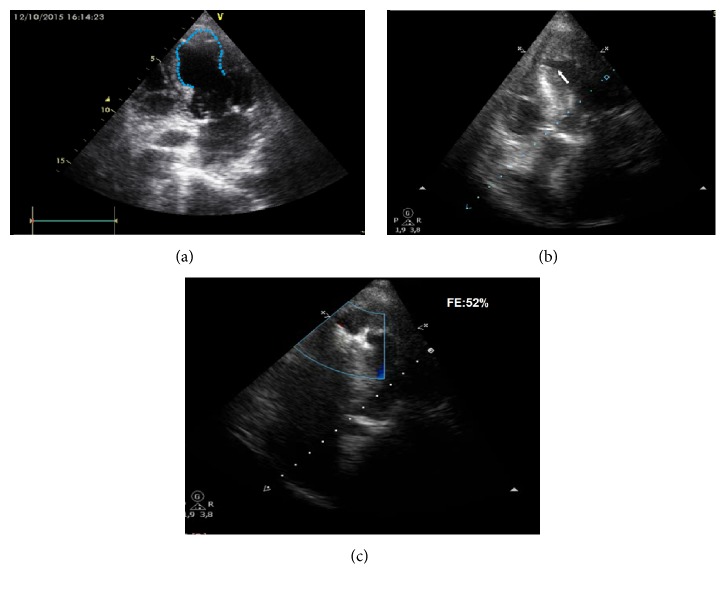
(a) Transthoracic echocardiogram (TTE) on admission, anterior and apical severe hypokinesia in both ventricles (apical ballooning pattern), and integrity of ventricular septum (dotted line and video); (b) TTE at the 4th day showing apical ventricular septal perforation (arrow) and a left ventricular ejection fraction of 41% (video); (c) TTE previous to discharge with the Amplatzer device and an improved left ejection fraction of 52%, with normal contractility in the right and left chambers (video).

On admission a transthoracic echocardiogram (TTE) showed a thin septum with a large area of hypokinesia in the apical, inferior apical, lateral apical, and anterior apical segments of the anterior left ventricular wall with an apical ballooning pattern (Figures 1 and 3) and a left ventricular ejection fraction of 41%.

**Figure 2:**
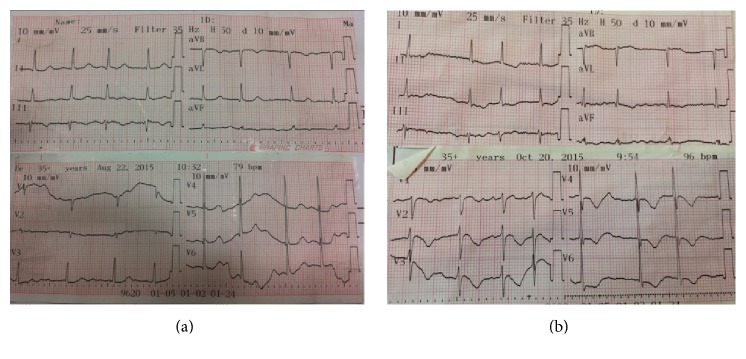
(a) Baseline EKG with no significant changes; (b) EKG with negative T waves in all precordial leads.

**Figure 3:**
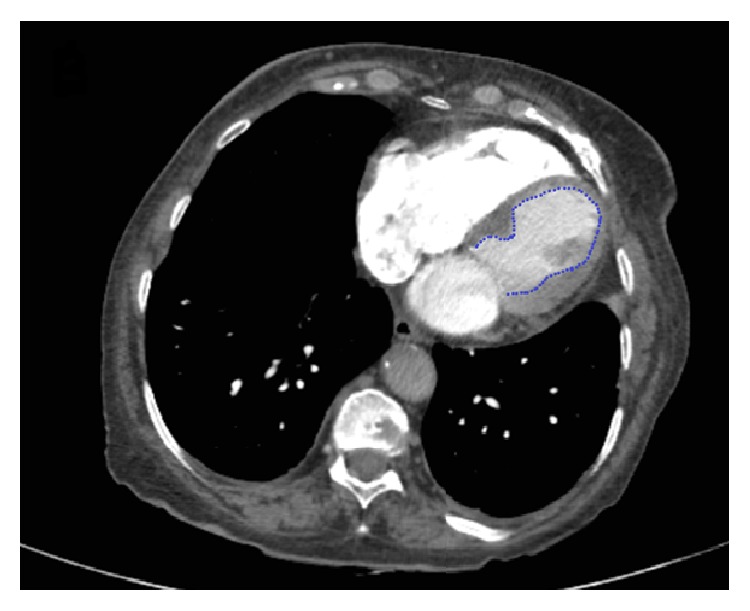
CT angiography showing the integrity of a thin ventricular septum with anterior and apical hypokinesia (apical ballooning pattern) of the left ventricle and also the right ventricle.

Right ventricular chambers revealed enlargement and hypokinesia (Figure 1(a), arrows) and the admission EKG showed no significant changes (Figure 2(a)). Six hours after admission cardiac enzymes were slightly increased, with a troponin of 116 *μ*g/L (upper limit of normal was 50) and a CK-MB of 5.6 *μ*g/L (upper limit of normal was 4.88). A cardiac tomography angiography was performed in order to rule out pulmonary embolism, which was normal, also confirming the integrity of a thin ventricular septum and the angiographic pattern of “apical ballooning” (Figure 3). In spite of the fact that after CT angiogram pulmonary embolism is discarded and the “apical ballooning” pattern suggested a presumptive diagnosis of TCM, coronary angiogram was deferred by reference physician preference and the patient was medically treated in a conservative manner.

At the 4th day of admission the patient presented symptoms and signs of congestive heart failure and a new onset of harsh, loud, and holosystolic murmur radiating to the back. In the EKG an inversion of T waves in all the precordial leads was observed (Figure 2).

**Figure 4:**
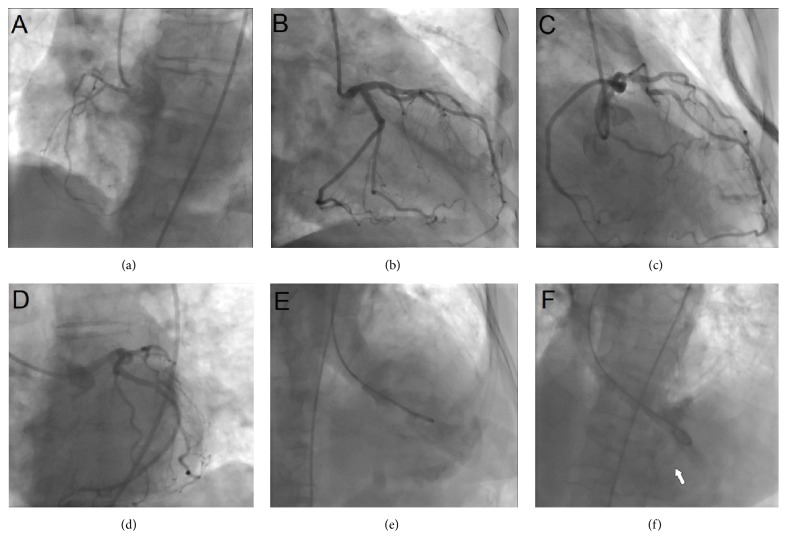
Coronary angiography describing coronary tree and biventricular function. (a) Small right coronary artery with an intermediate lesion in an acute marginal branch. ((b), (c), and (d)) Left circumflex and anterior descending coronary artery without significant lesions. ((e) and (f)) Left ventricular angiogram with the anterior and apical hypokinesia and the filling of right ventricle through the VSP (arrows).

A new TTE revealed similar compromise of the left and right apical walls of ventricles, but a VSP defect of 0.7/0.8 mm in apical portion of the septum was now observed, with the defect located 10 mm of distance from the apex of the left ventricle (Figure 1(b)). A coronary angiography was performed to assess the coronary tree. Right coronary artery was small without significant lesions and with a dominant left circumflex and left anterior descending (LAD) arteries “angiographically” normal; in the left ventriculogram, the septal defect was observed (Figure 4). The patient developed cardiogenic shock in spite of intensive medical treatment; therefore, a percutaneous procedure to close the VSP was planned.

**Figure 5:**
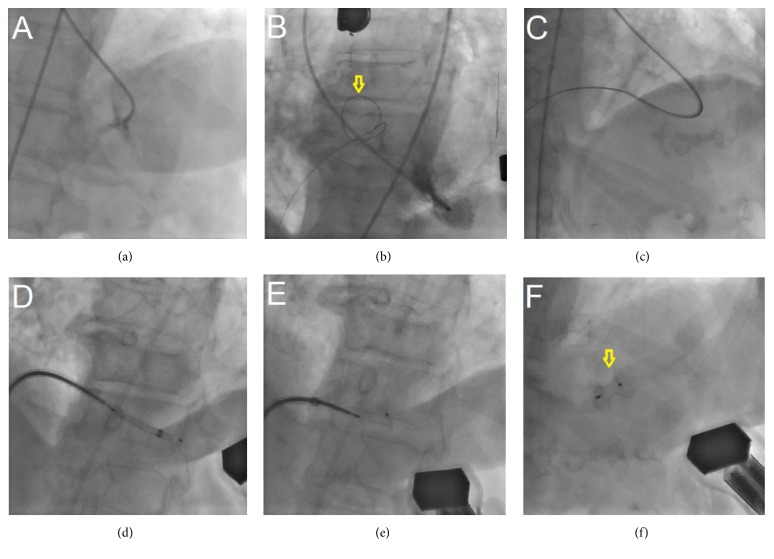
(a) Catheterization of the left ventricle and the ventricular septal perforation was achieved with an Amplatzer 1 guiding catheter; ((b) and (c)) guide wire deployed in pulmonary artery and the snore in the right atrium (arrow); ((d) and (e)) Amplatzer device previous implantation through the right to left ventricle; (f) Amplatzer device appropriately deployed (arrow).

At the 11th day of admission, the VSP was successfully repaired and closed percutaneously with an Amplatzer occluder device; the procedure was attempted using both femoral artery and vein access; fluoroscopy time of the procedure was 110 minutes (Figures 5(a)–5(f)).

Patient had had a significant improvement of functional class; TTE revealed recovery of right and left ventricular function (left ejection fraction, 52%) and good apposition of the Amplatzer device (Figure 1(c)).

Patient was discharged from hospital three days later and remained asymptomatic in our last contact two months after admission.

## 3. Discussion

TCM accounted for approximately 2% of all the patients with suspected acute coronary syndrome and 90% of these were postmenopausal women [1–3].

Our patient met all of major criteria for TCM: female at elderly age, extreme anxiety at admission, dyspnea, mild chest pain, slight elevation of cardiac enzymes, acute T waves changes in anterolateral leads, anterior and apical hypokinesia with hyperkinesia of basal segments, and no significant lesions in the coronary tree. Hypokinesia of the right ventricle observed on the admission TTE (Figure 1(a)) was an unusual finding but was previously reported in up to 40% of cases [5].

TCM was initially associated with good prognosis; however, severe complications including CR were also described. In a recent revision, CR was found in 14 cases including left and right free wall rupture in 10 cases and VSP in 4 cases [2, 4, 6, 7]; therefore, it should not be considered as a benign entity.

From these 4 cases, 2 survived after repair of the defect with conventional cardiac surgery and the other 2 died without treatment. Of interest, in such recent revision (2015), the authors did not take into consideration percutaneous technique as a potential tool to treat these patients [4]; in Table 1, we are describing treatment and outcome of these cases including ours.

In all percutaneous VSP closure procedures, access through femoral artery and femoral vein is mandatory.

First, a left catheterization of the left ventricle was done; the presence of an apical defect close to the apex increases the difficulty to appropriately reach the defect through left chambers and a wire guide was crossed from left to right ventricles and deployed into pulmonary artery. The guide wire was grabbed with a snare using the vein access allowing crossing the guiding catheter from right to left ventricles and the final deployment of Amplatzer device (Figure 5). The long fluoroscopy time in this case was related with the apical location of the defect in spite of the fact that most of the maneuvers to cross the defect were entirely guided by transesophageal echocardiography.

The only limitation of this presentation is that one cannot totally exclude the possibility that the patient actually had a coronary event such as a coronary embolus to the LAD with spontaneous lysis prior to angiography rather than TCM. However, the mild troponin elevation and biventricular presentation make this scenario, in our opinion, less likely. We also recognized that CT is not the best tool to assess left ventricular ballooning and cardiac magnetic resonance imaging (CMRI) is the best test to confirm diagnosis of TCM. This will help differentiate between myocardial oedema and myocardial scarring; however, by logistic reasons, CMRI was not available at the time of the rupture, and after the device was implanted, CMRI is contraindicated during the first three months after implantation.

In summary, to our knowledge, this is the first reported case using a percutaneous approach to repair this mechanical complication in a TCM.

## Supplementary Material

TEE before and after ventricular septal rupture (a and b) and after it was repaired with the Amplatzer device(c)

## Figures and Tables

**Table 1:** Treatment and clinical outcome in patients with takotsubo cardiomyopathy complicated with ventricular septal perforation.

Reference [4]	Case 1	Case 2	Case 3	Case 4	Case 5
Age	71	73	84	81	79
Cardiogenic shock	Yes	No	Yes	Yes	Yes
Left ventricular ejection fraction on admission (%)	25	49	Not done	67.2	41
Invasive treatment	Surgical repair	Surgical repair	None	None	Percutaneous closure with Amplatzer device
In-hospital outcome	Survival	Survival	Death	Death	Survival
